# Inhibition of Prostaglandin E2 Receptor EP3 Attenuates Oxidative Stress and Neuronal Apoptosis Partially by Modulating p38MAPK/FOXO3/Mul1/Mfn2 Pathway after Subarachnoid Hemorrhage in Rats

**DOI:** 10.1155/2022/7727616

**Published:** 2022-12-09

**Authors:** Yu Liu, Rui Liu, Lei Huang, Gang Zuo, Jiaxing Dai, Ling Gao, Hui Shi, Yuanjian Fang, Qin Lu, Takeshi Okada, Zhifei Wang, Xiao Hu, Cameron Lenahan, Jiping Tang, Jie Xiao, John H. Zhang

**Affiliations:** ^1^Department of Neurosurgery, The Third Xiangya Hospital of Central South University, 138 Tongzipo Road, Changsha, Hunan 410013, China; ^2^Department of Physiology and Pharmacology, School of Medicine, Loma Linda University, Loma Linda, CA 92350, USA; ^3^Department of Neurosurgery, Loma Linda University, Loma Linda, CA 92350, USA; ^4^Department of Emergency, The Third Xiangya Hospital of Central South University, 138 Tongzipo Road, Changsha, Hunan 410013, China; ^5^Department of Neurosurgery and Anesthesiology, Loma Linda University Medical Center, Loma Linda, CA 92350, USA

## Abstract

Oxidative stress and neuronal apoptosis contribute to pathological processes of early brain injury (EBI) after subarachnoid hemorrhage (SAH). Previous studies demonstrated that the inhibition of prostaglandin E2 receptor EP3 suppressed oxidative stress and apoptotic effects after Alzheimer's disease and intracerebral hemorrhage. This study is aimed at investigating the antioxidative stress and antiapoptotic effect of EP3 inhibition and the underlying mechanisms in a rat mode of SAH. A total of 263 Sprague–Dawley male rats were used. SAH was induced by endovascular perforation. Selective EP3 antagonist L798106 was administered intranasally at 1 h, 25 h, and 49 h after SAH induction. EP3 knockout CRISPR and FOXO3 activation CRISPR were administered intracerebroventricularly at 48 h prior to SAH, while selective EP3 agonist sulprostone was administered at 1 h prior to SAH. SAH grade, neurological deficits, western blots, immunofluorescence staining, Fluoro-Jade C staining, TUNEL staining, 8-OHdG staining, and Nissl staining were conducted after SAH. The expression of endogenous PGES2 increased and peaked at 12 h while the expression of EP1, EP2, EP3, EP4, and Mul1 increased and peaked at 24 h in the ipsilateral brain after SAH. EP3 was expressed mainly in neurons. The inhibition of EP3 with L798106 or EP3 KO CRISPR ameliorated the neurological impairments, brain tissue oxidative stress, and neuronal apoptosis after SAH. To examine potential downstream mediators of EP3, we examined the effect of the increased expression of activated FOXO3 following the administration of FOXO3 activation CRISPR. Mechanism studies demonstrated that L798106 treatment significantly decreased the expression of EP3, p-p38, p-FOXO3, Mul1, 4-HNE, Bax, and cleaved caspase-3 but upregulated the expression of Mfn2 and Bcl-2 in SAH rats. EP3 agonist sulprostone or FOXO3 activation CRISPR abolished the neuroprotective effects of L798106 and its regulation on expression of p38MAPK/FOXO3/Mul1/Mfn2 in the ipsilateral brain after SAH. In conclusion, the inhibition of EP3 by L798106 attenuated oxidative stress and neuronal apoptosis partly through p38MAPK/FOXO3/Mul1/Mfn2 pathway post-SAH in rats. EP3 may serve as a potential therapeutic target for SAH patients.

## 1. Background

Aneurysmal subarachnoid hemorrhage (SAH) is a life-threatening cerebrovascular disease associated with a high incidence of mortality and morbidity [1–5]. Early brain injury (EBI) is an important pathological process that causes the poor prognosis in patients after SAH [6–8]. Recent studies reported that the oxidative stress and mitochondria-mediated neuronal apoptosis both play critical roles in the pathogenesis of EBI [9–13]. Thus, effective treatment that attenuates oxidative stress and neuronal apoptosis would improve the neurological outcomes in SAH patients.

Prostaglandin E2 (PGE2), the most widely produced prostanoid in the human body, is responsible for distinct biological outcomes via four specific G-protein-coupled receptors: prostaglandin E receptor (EP)1, EP2, EP3, and EP4 [14]. Each of these receptors differs in their tissue localization, expression regulation mechanisms, and signal transduction pathways. Among the four PGE2 receptors, EP3 is the most abundant EP receptor in the brain, which is presented on the plasma membrane and the cell nuclei membranes [15–17]. In the central nervous system (CNS), EP3 inhibition abrogated the migration and proliferation of human glioblastoma cells [18], alleviated cognitive deficits in the APP/PS1 mouse model of AD [19], attenuated brain injury, and improved neurological functional recovery in ischemic stroke model of mice [20]. The recent research has demonstrated that knocking down EP3 by RNA interference attenuated neuronal apoptosis through decreasing expression of active caspase-3 and proapoptotic Bcl-2-associated X protein (Bax) and increasing expression of antiapoptotic protein B cell lymphoma-2 (Bcl-2) in vivo and in vitro after the experimental ICH [21]. The highly selective EP3 receptor antagonist (N-[(5-bromo-2-methoxyphenyl)sulfonyl]-3-[2-(2-naphthalenylmethyl)phenyl]-2-propenamide, L798106) suppressed inflammation and improved glucose insulin tolerance in a mice model of diabetic dyslipidemia [22]. L798106 administration abrogated oxidative stress along with neuronal nitric oxide synthase phosphorylation in the animal model of central cardiovascular regulation via EP3 pathway [23]. However, the effects of EP3 inhibition on EBI remains largely unknown after SAH.

Mitochondrial dysfunction was closely related to oxidative stress response and neuronal apoptosis in numerous neurological disorders [10, 24–28]. Targeting mitochondrial dysfunction therefore appears to be an effective therapeutic strategy for SAH [29–31]. Mitochondrial E3 ubiquitin ligase 1 (Mul1) is a mitochondrial membrane protein which has effects on cell growth, stimulation of apoptosis, and modulation of antiviral signaling in innate immunity [32]. Mitofusin2 (Mfn2) is a GTPase embedded in the outer membrane of the mitochondria which can mediate mitochondrial fusion. The downregulation of Mfn2 by Mul1 increased the fragmented mitochondria concomitant with the mitochondrial dysfunction and cell deaths after the experimental ischemic stroke [33]. Forkhead box O3 (FOXO3), a member of the FOXO transcription factor subfamily, emerges as a versatile target for diseases that impact upon neuronal survival, vascular integrity, immune function, and cellular metabolism [34]. Interestingly, FOXO3 activation could increase the transcription of Mul1, which ubiquitinates and degrades Mfn2 and therefore leads to mitochondrial fragmentation in differentiated skeletal muscle [35]. It is reported that phosphorylation of p38MAPK leaded to the activation of FOXO3 and the subsequent induction of apoptosis and inhibition of cell proliferation [36]. PGE2/EP3/p38MAPK/Bcl-2 signaling pathway promoted endothelial apoptosis in cultured human umbilical vein endothelial cells [37]. Thus, it is likely that Mul1/Mfn2 may involve in the EP3-mediated apoptosis and oxidative stress following the brain injury.

Collectively, we hypothesize that L798106 will inhibit oxidative stress injury and neuronal apoptosis through inhibiting EP3/p38MAPK/FOXO3/Mul1/Mfn2 pathway after SAH in rats.

## 2. Materials and Methods

### 2.1. Animals

Two hundred and sixty-three adult male Sprague–Dawley (SD) rats (280–320 g, Indianapolis, IN, USA) were housed in a room with constant temperature (22 ± 1°C), humidity control (60 ± 5%), 12 h day/night, and unlimited water and food. The experimental protocol was approved by the Institutional Animal Care and Use Committee (IACUC) at Loma Linda University (No. 20-009). All experiments strictly followed the National Institutes of Health's Guide for the Care and Use of Laboratory Animals and the ARRIVE guidelines (Animal Research: Reporting In Vivo Experiments).

### 2.2. SAH Model

The SAH endovascular perforation model was induced as previously described [38]. Briefly, under 5% isoflurane anesthesia, the rats were intubated and ventilated with 2.5% isoflurane in mixed medical air and oxygen gas. After rats were placed in a supine position, the left carotid artery and its bifurcation were exposed. A 4-0 nylon sharpened suture was inserted into the left external carotid artery and advanced through the internal carotid artery to reach the bifurcation of the middle and anterior cerebral artery. Afterwards, the suture was further advanced 3 mm to puncture the bifurcation of artery. Rats in the sham group underwent the same surgery except for the perforation of blood vessel. After the surgery, the rat was extubated and observed in a recovery cage until fully recovered from anesthesia.

### 2.3. SAH Grading

The evaluation of SAH grading score was conducted at 24 h after SAH induction as previously published by an investigator blind to group information [39]. In general, the basal cistern, including the brain stem, was divided into six parts, each graded from 0 to 3 according to the blood volume. The total score was calculated by adding all area scores (ranging from 0 to 18). Rats with mild SAH (SAH grading score ≤ 8) were excluded from the current study.

### 2.4. Experimental Design

The current study was performed in four separate experiments, as illustrated in (Fig. s1).

#### 2.4.1. Experiment 1: Time Course of PGE2, EP1, EP2, EP3, EP4, and Mul1 and Cellular Localization of EP3 in Male Rats after SAH

Thirty-six adult male SD rats were randomly divided into six groups (*n* = 6 per group): sham and SAH (3, 6, 12, 24, and 72 h). The protein levels of PGE2, EP1, EP2, EP3, EP4, and Mul1 in the ipsilateral brain hemisphere were evaluated by western blot. PGES2 catalyzes the conversion of PGH2 into more stable PGE2. The level of isomerase is an indirect indicator of PGE2 level in brain tissue. In this study, PGES2 protein level was measured as a proxy for PEG2 level. The double immunofluorescence staining was performed in the sham group and SAH (24 h) group (*n* = 2 per group) to evaluate the cellular localization of EP3.

#### 2.4.2. Experiment 2: The Effect of EP3 Inhibition on Short-Term Outcomes in Male Rats after SAH

Forty-two adult male SD rats were randomized to seven groups (*n* = 6 per group): sham, SAH + vehicle (10% dimethyl sulfide), SAH + L798106 (60 *μ*g/kg), SAH + L798106 (180 *μ*g/kg), SAH + L798106 (540 *μ*g/kg), SAH + scramble CRISPR, and SAH + EP3 knockout (KO) CRISPR. The selective EP3 inhibitor L798106 was administrated intranasally (i.n.) 1 h after SAH. CRISPR was administered intracerebroventricularly (i.c.v.) 48 h prior to SAH induction. Neurological test and SAH grade were accessed at 24 h after SAH. Based on the results of short-term neurological test, the optimal dosage of 180 *μ*g/kg was chosen for the oxidative stress/neuronal apoptosis assessment, long-term neurological function, and mechanism study. Oxidative stress and neuronal apoptosis/degeneration were evaluated by the brain tissue staining of 8-OHdG, terminal deoxynucleotidyl transferase dUTP-biotin nick end labeling (TUNEL), and Fluoro-Jade C at 24 h after SAH. Additional eighteen rats (*n* = 6 per group) were randomized into three groups including sham, SAH + vehicle (10% dimethyl sulfide), and SAH + L798106 (180 *μ*g/kg), which were used for evaluating neuronal apoptosis/neuronal degeneration at 7 d after SAH.

#### 2.4.3. Experiment 3: The Effect of EP3 Inhibition on Long-Term Outcomes in Male Rats after SAH

Thirty adult male SD rats were randomly distributed to three groups (*n* = 10 per group): sham, SAH + vehicle (10% dimethyl sulfide), and SAH + L798106 for evaluating the long-term neurological functions and histopathology. The rotarod test was conducted to evaluate the motor skills and coordination ability on days 7, 14, and 21 after SAH. Morris water maze test was carried out during days 22-27 after SAH. Brain samples were collected on the 28 days after SAH to evaluate the neuronal degeneration using Nissl staining.

#### 2.4.4. Experiment 4: Mechanism Study

To investigate the potential molecular mechanism of EP3 inhibition, forty-two adult male SD rats were randomly divided to seven groups (*n* = 6 per group): sham, SAH + vehicle (10% dimethyl sulfide, i.n.), SAH + L798106 + scrambled CRISPR, SAH + L798106, SAH + L798106 + FOXO3 activation CRISPR, SAH + L798106, and SAH + L798106 + sulprostone. To validate the efficacy of the CRISPR/Cas9-mediated knock-in or knockout, third-six more rats (*n* = 6 per group) were assigned to six groups: naïve + scramble CRISPR, naive + EP3 KO CRISPR, naive + FOXO3 CRISPR, SAH + scramble CRISPR, SAH + EP3 KO CRISPR, and SAH + FOXO3 activation CRISPR. L798106/vehicle was administrated i.n. 1 h after SAH. CRISPR or sulprostone/vehicle was administered intracerebroventricularly (i.c.v.) in rats at 48 h or 1 h prior to SAH induction. Western blotting was performed using ipsilateral brain hemisphere at 24 h after SAH induction or 24 h after CRISPR injection in naïve rats.

### 2.5. Drug Administration

#### 2.5.1. Intranasal Drug Administration

The administration of drugs by the intranasal route was performed 1 h after SAH induction [40]. A total volume of 30 *μ*l vehicle (10% dimethyl sulfide, W274623, Sigma-Aldrich, USA) or L798106 (Santa Cruz Biotechnology, Dallas, USA) at three different dosages (60 *μ*g/kg, 180 *μ*g/kg, and 540 *μ*g/kg) was delivered alternately into the bilateral nares. The dose of L798106 treatment was chosen based on a recent study [41].

#### 2.5.2. Intracerebroventricular Drug Administration

An intracerebroventricular injection was conducted as described before [42]. Animals were kept in a stereotaxic apparatus with 3% isoflurane mixed air (70% medical air/30% oxygen) anesthesia. A burr hole was drilled on the skull at the following coordinates relative to bregma: 1.5 mm posterior and 1.0 mm lateral. The needle of a 10 *μ*l Hamilton syringe (Microliter 701, Hamilton Company, Reno, NV, USA) was inserted through the burr hole into the left lateral ventricle at a depth of 3.3 mm. The speed of infusion was maintained at 1 *μ*l/min using an infusion pump (Stoelting, Harvard Apparatus, Holliston, MA, USA). A total of 2 *μ*g knockout or activation CRISPR was delivered intracerebroventricularly (i.c.v). EP3 KO CRISPR (sc-422482, Santa Cruz Biotechnology, Dallas, TX, USA), FOXO3 activation CRISPR (sc-425192-ACT, Santa Cruz Biotechnology, Dallas, TX, USA), or scramble CRISPR (sc-418922, Santa Cruz Biotechnology, Dallas, TX, USA) was injected into the cerebroventricle 48 h prior to SAH induction.

### 2.6. Neurological Performance

These neurological functions were evaluated by two independent investigators blindly to the experimental groups.

#### 2.6.1. Short-Term Neurological Performance

The modified Garcia test and the beam balance test were conducted to evaluate short-term neurological deficits at 24 h post-SAH [12]. The modified Garcia score consisted of 6 categories and ranging from 3 to 18 points. The beam balance tests investigated the ability of rats to maintain balance when walking on an elevated beam for 60 seconds. The score ranged from 0 to 4 points.

#### 2.6.2. Long-Term Neurological Performance

*(1) Rotarod Test*. Rotarod test was conducted to assess the motor impairment of animals at weeks 1, 2, and 3 after SAH induction [43, 44]. The rotating speed starts at 5 revolutions per minute (RPM) and 10 RPM, and the speed increased 2 RPM every 5 s. The durations of rats on the rotarod were recorded.

*(2) Morris Water Maze*. Morris water maze was performed to assess the memory and spatial learning abilities on days 22-27 after SAH induction [45, 46]. In general, the animals were tested for the capability to find platform in 60 seconds. On the last day, the platform was removed, and the rats were tested for the time spent in platform quadrant in 60 seconds. A video recording system traced the rats' activities. The animals' swim paths were analyzed for quantifying swimming distance, latency to reach the platform/time spent in the platform quadrant, and swimming speed by Video Tracking System SMART-2000 (San Diego Instruments Inc., San Diego, CA).

### 2.7. Immunofluorescence Staining

Immunofluorescence staining was performed as previously described [47, 48]. Briefly, the animals were perfused with ice cold PBS and followed by 10% formalin under deep anesthesia. The animals' brains were further fixed in 10% formalin at 4°C for 48 h and dehydrated in 30% sucrose solution for a week. The brains were sectioned to 10 *μ*m thick slices. The brain slices were stained overnight with following primary antibodies including rabbit anti-EP3 (1 : 50, 14357-1-AP, Proteintech, Rosemont, USA), mouse anti-NeuN (1 : 100, ab177487, Abcam, MA, USA), mouse anti-Iba1 (1 : 100, ab15690, Abcam, MA, USA), and mouse anti-GFAP (1 : 100, ab7260, Abcam, MA, USA) overnight at 4°C. On the second day, the slices were incubated with the respective fluorescence dye-conjugated secondary antibodies (1 : 200, Jackson ImmunoResearch, PA, USA) on dark condition for 2 h followed by DAPI staining. The staining was observed using a fluorescence microscope (Olympus, Melville, NY, USA).

### 2.8. Fluoro-Jade C Staining

Fluoro-Jade C (FJC) staining was conducted to assess the number of degenerating neurons post-SAH as previously described [49]. FJC Ready-to-Dilute Staining Kit (Biosensis, USA) was used in accordance with the manufacturer's instruction. The staining were observed using a fluorescence microscope (Olympus, Melville, NY, USA), and microphotographs were taken under ×200 magnification. The FJC-positive neurons were counted within ipsilateral cortex in 6 random slices from 1.7 mm anterior to bregma through 4.3 mm posterior to bregma per animal using ImageJ software (ImageJ 1.5, NIH, USA). The data were calculated and presented as the average number of FJC-positive neurons per mm^2^.

### 2.9. TUNEL Staining

Double immunofluorescence staining of terminal deoxynucleotidyl transferase dUTP nick end labeling (TUNEL) and neuron marker NeuN was applied to assess the neuronal damage post-SAH. The staining was observed using a fluorescence microscope (Olympus, Melville, NY, USA), and microphotographs were taken under ×200 magnification. The TUNEL-positive neurons were counted within ipsilateral cortex of six random brain slices from 1.7 mm anterior to bregma through 4.3 mm posterior to bregma per animal per animal using ImageJ software (ImageJ 1.5, NIH, USA). The result data was presented as the percentage of TUNEL-positive neurons (%) in the total number of neurons.

### 2.10. 8-OHdG

To evaluate the oxidative stress and mitochondrial ROS level, the staining of 8-OHdG was performed as previously reported [7]. Briefly, after the antigen retrieval and endogenous peroxidase activity blockage, the brain slices were incubated with 8-hydroxy-2′-deoxyguanosine (8-OHdG) antibody (1 : 200, ab62623, Abcam, Cambridge, MA, USA) at room temperature. Six random slices from 1.7 mm anterior to bregma through 4.3 mm posterior to bregma were visualized under a fluorescence microscope, and microphotographs were taken under ×200 magnification. The relative fluorescence intensity within ipsilateral cortex was analyzed by ImageJ software (ImageJ 1.5, NIH, USA).

### 2.11. Nissl Staining

Nissl staining was conducted on 15 *μ*m thick brain slices to assess hippocampal neuron degeneration as previously reported [50]. The staining of six random slices from 1.7 mm anterior to bregma through 4.3 mm posterior to bregma was visualized under a fluorescence microscope, and microphotographs were taken under ×200 magnification. The average number of survival neurons in the hippocampus CA1, CA3, and dentate gyrus (DG) area was calculated.

### 2.12. Western Blot Analysis

Western blot was conducted as previously reported [51]. The ipsilateral (left) cerebral hemispheres were homogenized in RIPA buffer (Santa Cruz Biotechnology, CA, USA) and centrifuged at 14,000 rpm for 30 minutes. After the determination of protein concentration, equal protein sample was separated by 10% SDS-PAGE gel and transferred to nitrocellulose membranes (0.22 or 0.45 *μ*m). Membranes were incubated overnight at 4°C with the following primary antibodies: anti-PGE2 (1 : 1000, BS-2639R, Bioss Antibodies Inc., Massachusetts, USA), anti-EP3 (1 : 1000, 14357-1-AP, Proteintech, Rosemont, USA), anti-p38 (1 : 1000, 9212S, Cell Signaling Technology Inc., MA, USA), anti-p-p38 (1 : 1000, 9211S, Cell Signaling Technology Inc., MA, USA), anti-FOXO3 (1 : 1000, MA5-14932, Life Technologies Corporation, NY, USA), anti-p-FOXO3 (Ser7) (1 : 1000, 14724S Cell Signaling Technology Inc., MA, USA), anti-Mul1 (1 : 1000, 16133-1-AP, Proteintech, Rosemont, USA), anti-Mfn2 (1 : 1000, MA5-27647, Life Technologies Corporation, NY, USA), anti-Bcl-2 (1 : 1500, ab59348, Abcam, MA, USA), anti-Bax (1 : 1500, ab182734, Abcam, MA, USA), anti-4-HNE (1 : 1500, ab46545, Abcam, MA, USA), anti-cleaved caspase-3 (1 : 1000, D175, Cell Signaling Technology, MA, USA), and anti-*β*-tubulin (1 : 500, ab6046, Abcam, MA, USA). On the next day, the membranes were incubated with the secondary antibody at room temperature for 2 h. The relative density of protein bands was quantified with the ImageJ (ImageJ 1.5, NIH, USA). The results were normalized to their own tubulin of the same sample.

### 2.13. Statistical Analysis

All data were presented as the mean and standard deviation (mean ± SD). GraphPad Prism (Graph Pad Software, San Diego, CA, USA) was applied to analyze the data. The Kolmogorov-Smirnov (K-S) test was used for normality. Data in each experiment passed K-S test allowed for parametric tests. One-way ANOVA followed by Tukey's post hoc test was performed for multiple comparisons among different groups. The results of long-term neurological performance were analyzed using two-way ANOVA. *p* < 0.05 was considered statistically significant.

## 3. Results

### 3.1. Mortality and SAH Grade

**Figure 1:**
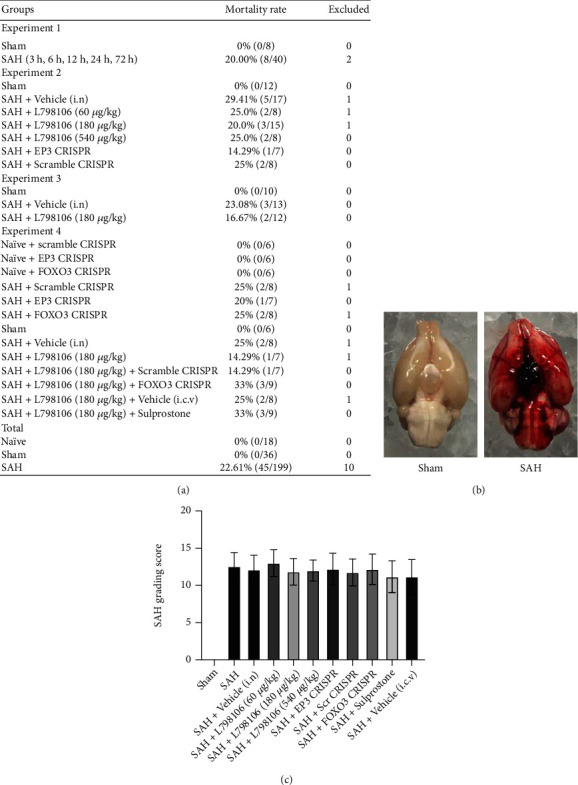
Mortality rate and subarachnoid hemorrhage (SAH) grade. (a) The number of mortality and excluded animals in each group. (b) Representative image of SAH model in rats. (c) SAH grade in each group. Vehicle, 10% dimethyl sulfoxide (DMSO).

A total of 263 rats were used in the present study, of which 36 rats assigned to the sham group, 18 assigned to the naïve group, and 199 subjected to the SAH group (Figure 1(a)). The mortality was 22.61% (45/199) in SAH rats. None of sham animals (0/36) or naïve animals (0/18) died. Blood clots were mainly distributed around the circle of Willis and ventral brain stem after SAH induction (Figure 1(b)). There were no significant differences in SAH grading scores among the SAH groups (Figure 1(c)). Nine rats were excluded from this study due to mild SAH.

### 3.2. Expression of Endogenous PGE2, EP1, EP2, EP3, EP4, and Mul1 after SAH

**Figure 2:**
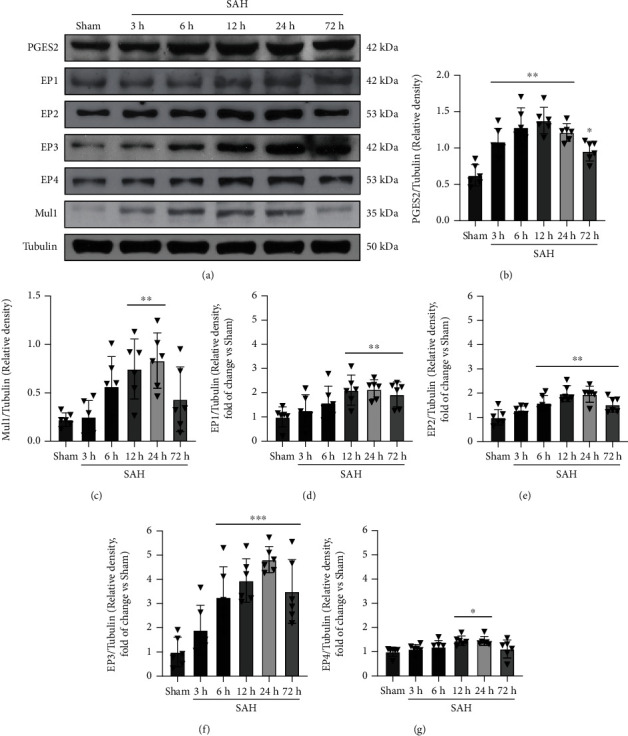
Time course of PGES2, EP1, EP2, EP3, EP4, and Mul1 expression in the ipsilateral hemisphere brain after subarachnoid hemorrhage (SAH). (a) Representative western blot images and densitometric quantification of PGES2 (b), Mul1 (c), EP1 (d), EP2 (e), EP3 (f), and EP4 (g) after SAH. ^∗^*p* < 0.05, ^∗∗^*p* < 0.01, and ^∗∗∗^*p* < 0.001 vs. sham group. Data was expressed as mean ± SD, *n* = 6 per group, one-way ANOVA-Tukey.

The time course of brain endogenous PGES2, 4 subtypes of PGE2 receptors (EP1-4), and Mul1 expression were evaluated by western blot. The endogenous ligand PGES2 expression was increased rapidly within 3 h and peaked at 12 h post-SAH induction, suggesting an increase in PGE2 ligand. The protein levels of 4 subtypes of PGE2 receptors were markedly increased at 6 h after SAH and peaked around 24 h, of which the extent of EP3 increase was the most. The expression of Mul1, a potential downstream signal protein, was also upregulated in a similar pattern to EP3 (Figure 2).

**Figure 3:**
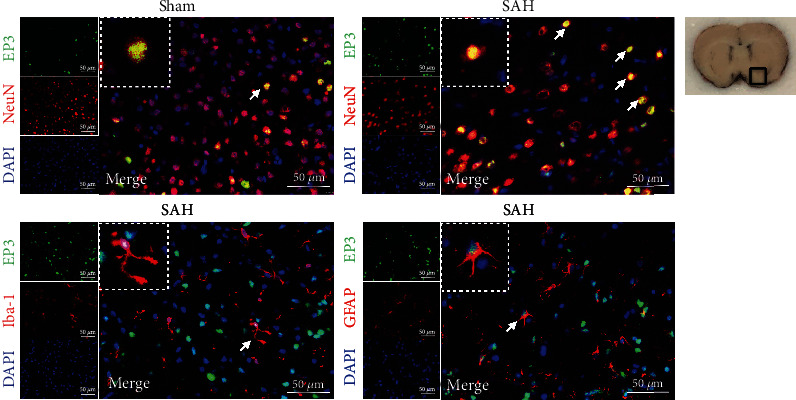
Double immunofluorescence staining of EP3 with NeuN, GFAP, and Iba-1 at 24 h after SAH. Top panel indicates the location of staining (small black box). Scale bar = 50 *μ*m. *n* = 2 for each group.

Double immunofluorescence staining of EP3 with NeuN (a marker for neurons), GFAP (a marker for astrocytes), or Iba-1 (a marker for microglia) was conducted in the sham group and 24 h SAH group. The results revealed that EP3 receptor was mainly located in neurons, some in microglia and astrocytes in the brain. There was a significantly greater number of EP3-positive neurons in SAH rats than shams (Figure 3).

### 3.3. EP3 Inhibition Reduced Short-Term Neurological Deficits at 24 h after SAH

**Figure 4:**
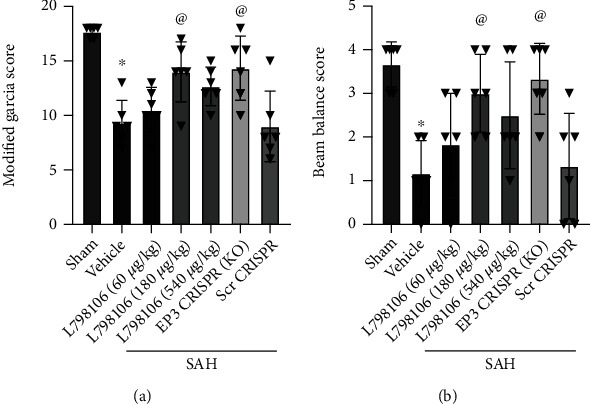
EP3 inhibition improved short-term (24 h) neurological outcome after SAH. L798106 and EP3 CRISPR improved the Modified Garcia score (a) and beam balance score (b) at 24 hours after SAH. Vehicle: 10% DMSO. ^∗^*p* < 0.05 vs. sham group; ^@^*p* < 0.05 vs. SAH + vehicle group. Data was expressed as mean ± SD, *n* = 6 per group, one-way ANOVA-Tukey.

The score of the modified Garcia and beam balance tests were significantly decreased in the SAH + vehicle group at 24 h post-SAH when compared to the sham group (Figure 4). EP3 KO CRISPR and L798106 treatments at a dosage of 180 *μ*g/kg significantly improved the modified Garcia score and beam balance score compared with the SAH + vehicle group at 24 h after SAH (Figure 4). Based on the results of short-term neurological function, the dosage of 180 *μ*g/kg was chosen as the best dosage of L798106 in other experiments of this study.

### 3.4. EP3 Inhibition Reduced Neuronal Apoptosis and Neuronal Degeneration at 24 h after SAH

**Figure 5:**
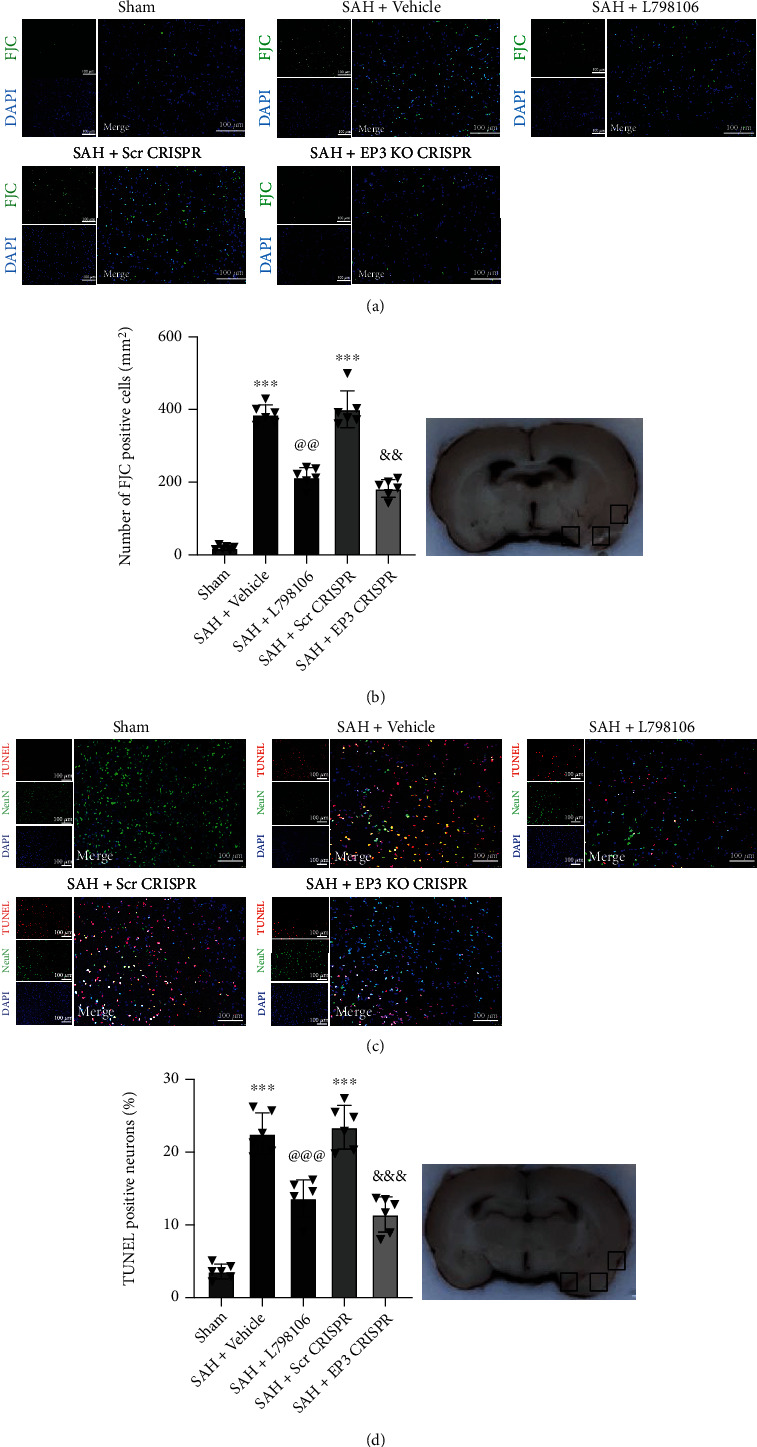
Effects of EP3 inhibition on short-term (24 h) neuronal degeneration and apoptosis after SAH. (a) Representative microphotographs of FJC immunofluorescence staining in the ipsilateral cortex of rat brain. (b) Quantitative analysis of FJC-positive cells. Top panel indicates the location of staining (small black box). (c) Representative microphotographs of TUNEL immunofluorescence staining in the ipsilateral cortex of rat brain. (d) Quantitative analysis of TUNEL-positive neurons. Top panel indicates the location of staining (small black box). Vehicle: 10% DMSO. ^∗∗∗^*p* < 0.001 vs. sham group; ^@@^*p* < 0.01 and ^@@@^*p* < 0.001 vs. SAH + vehicle group; ^&&^*p* < 0.01 and ^&&&^*p* < 0.001 vs. SAH + Scr CRISPR group. Scale bar = 100 *μ*m. Data was expressed as mean ± SD, *n* = 6 per group, one-way ANOVA-Tukey.

FJC staining and TUNEL staining were conducted to assess neuronal degeneration and apoptosis in the ipsilateral cerebral cortex. There was a significant increase in FJC-positive and TUNEL-positive neurons in SAH rats at 24 h post-SAH, which were significantly reduced by EP3 KO CRISPR or L798106 (Figure 5).

### 3.5. EP3 Inhibition Reduced Brain Oxidative Stress Injury at 24 h after SAH

**Figure 6:**
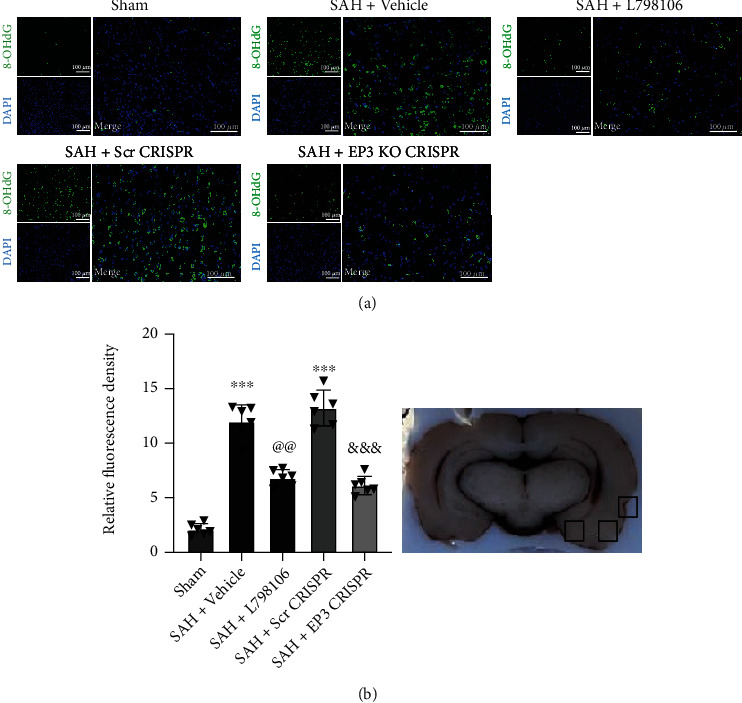
Effects of EP3 inhibition on short-term (24 h) oxidative stress level after SAH. (a) Representative microphotographs of 8-OHdG immunofluorescence staining in the ipsilateral cortex of rat brain. (b) Quantitative analysis of 8-OHdG fluorescence intensity (*n* = 6 per group). Top panel indicates the location of staining (small black box). Vehicle: 10% DMSO. ^∗∗∗^*p* < 0.001 vs. sham group; ^@@^*p* < 0.01 vs. SAH + vehicle group; ^&&&^*p* < 0.001 vs. SAH + Scr CRISPR group. Scale bar = 100 *μ*m. Data was expressed as mean ± SD, one-way ANOVA-Tukey.

The immunofluorescence staining of 8-OHdG was conducted to assess the oxidative stress damage in the ipsilateral cerebral cortex. SAH resulted in a significant increase in fluorescence intensities of 8-OHdGs at 24 h post-SAH, which was significantly reduced by EP3 KO CRISPR or L798106 (Figure 6).

### 3.6. EP3 Inhibition with L798106 Reduced Neuronal Apoptosis and Neuronal Degeneration at 7 d after SAH

**Figure 7:**
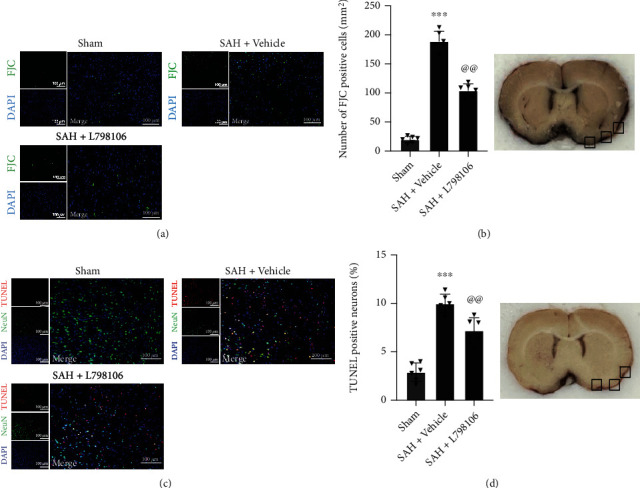
Effects of EP3 inhibition with L798106 on neuronal degeneration and apoptosis at 7 d after SAH. (a) Representative microphotographs of FJC immunofluorescence staining in the ipsilateral cortex of rat brain. (b) Quantitative analysis of FJC-positive cells. Top panel indicates the location of staining (small black box). (c) Representative microphotographs of TUNEL immunofluorescence staining in the ipsilateral cortex of rat brain. (d) Quantitative analysis of TUNEL-positive neurons. Top panel indicates the location of staining (small black box). Vehicle: 10% DMSO. ^∗∗∗^*p* < 0.001 vs. sham group; ^@@^*p* < 0.01 vs. SAH + vehicle group. Scale bar = 100 *μ*m. Data was expressed as mean ± SD, *n* = 6 per group, one-way ANOVA-Tukey.

FJC staining and TUNEL staining were conducted to assess the neuronal degeneration and neuronal apoptosis in the ipsilateral cerebral cortex at 7 d post-SAH. SAH resulted in a significant increase in FJC-positive and TUNEL-positive neurons at 7 d post-SAH, which were significantly reduced by L798106 treatment (Figure 7).

### 3.7. EP3 Inhibition with L798106 Improved Long-Term Neurological Deficits at 28 d after SAH

**Figure 8:**
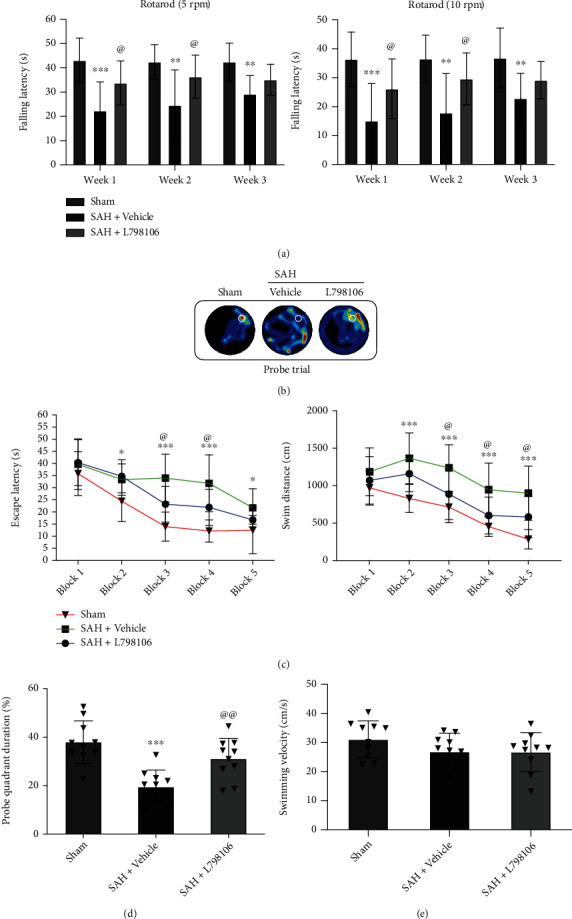
Effects of EP3 inhibition with L798106 on long-term (28 d) neurobehavioral outcome after SAH. (a) L798106 increased falling latency in rotarod test on day 7s and 14 after SAH. (b) Representative thermal imaging of the probe trial. The white circles indicate the positions of the probe platform. (c) Escape latency and swimming distance of Morris water maze. (d) Quantification of the probe quadrant duration in the probe trial. (e) Swimming velocities of different groups in probe trial. Vehicle: 10% DMSO. ^∗^*p* < 0.05, ^∗∗^*p* < 0.01, and ^∗∗∗^*p* < 0.001 vs. sham group; ^@^*p* < 0.05 and ^@@^*p* < 0.01 vs. SAH + vehicle group. Data was expressed as mean ± SD, *n* = 10 per group, group; two-way ANOVA-Tukey (a, c) and one-way ANOVA-Tukey (d, e).

In the rotarod test, the SAH rats had a markedly shorter falling latency compared to the shams at days 7, 14, and 21 post-SAH. L798106 treatment improved the rotarod performance in SAH rats at 7 and 14 days but not at 21 days post-SAH (Figure 8(a)).

Morris water maze test showed that SAH rats had a longer escape latency to find the platform, more swimming distances (Figure 8(c)) and less time spent in target quadrant (Figure 8(d)) at 22-27 d after SAH, suggesting the impaired spatial learning memory. L798106 treatment significantly improved the performance of Morris water maze test in SAH rats (Figures 8(c) and 8(d)). There was no statistical difference in swimming speed among all three groups (Figure 8(e)).

### 3.8. EP3 Inhibition with L798106 Reduced Neuronal Degeneration at 28 d after SAH

**Figure 9:**
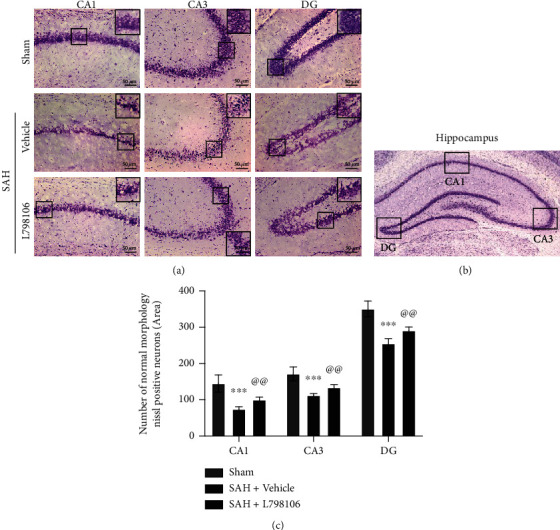
Effects of EP3 inhibition with L798106 on long-term (28 d) neuronal degeneration after SAH. (a) Representative microphotographs of Nissl staining in different hippocampal regions. Scale bar = 50 *μ*m. (b) Representative image indicates the location of interest area (small black boxes) in the left hippocampus. (c) Quantification of Nissl-positive neurons per area in dentate gyrus (DG), cornu ammonis (CA1), and CA3 at 28 days after SAH. ^∗∗∗^*p* < 0.001 vs. sham group and ^@@^*p* < 0.01 vs. SAH + vehicle group. Data were expressed as mean ± SD, *n* = 10 per group. One-way ANOVA-Tukey.

Nissl staining was conducted to assess the neuronal degeneration of hippocampus area at 28 d post-SAH. The number of survival neurons in hippocampus CA1, CA3, and DG regions in the SAH + vehicle group was markedly lower than that in the sham group. L798106 treatment significantly preserved hippocampal neuronal survival in CA1, CA3, and DG regions in SAH rats (Figure 9).

### 3.9. Efficacy of EP3 Knockout CRISPR and FOXO3 Activation CRISPR

The i.c.v. injection of EP3 KO CRISPR at 48 h prior to SAH or in naive rats markedly downregulated the protein expression of EP3 in SAH rats or naive rats. The i.c.v injection of FOXO3 ACT CRISPR at 48 h prior to SAH or in naive rats remarkably increased the protein expression of p-FOXO3 in SAH rats or naive rats. The results demonstrated knockout and activate efficiencies of EP3 or FOXO3 CRISPRs (Fig. s2).

### 3.10. EP3 Inhibition with L798106 Attenuated Neuronal Apoptosis and Oxidative Stress via p38MAPK/FOXO3/Mul1/Mfn2 Pathway at 24 h after SAH

**Figure 10:**
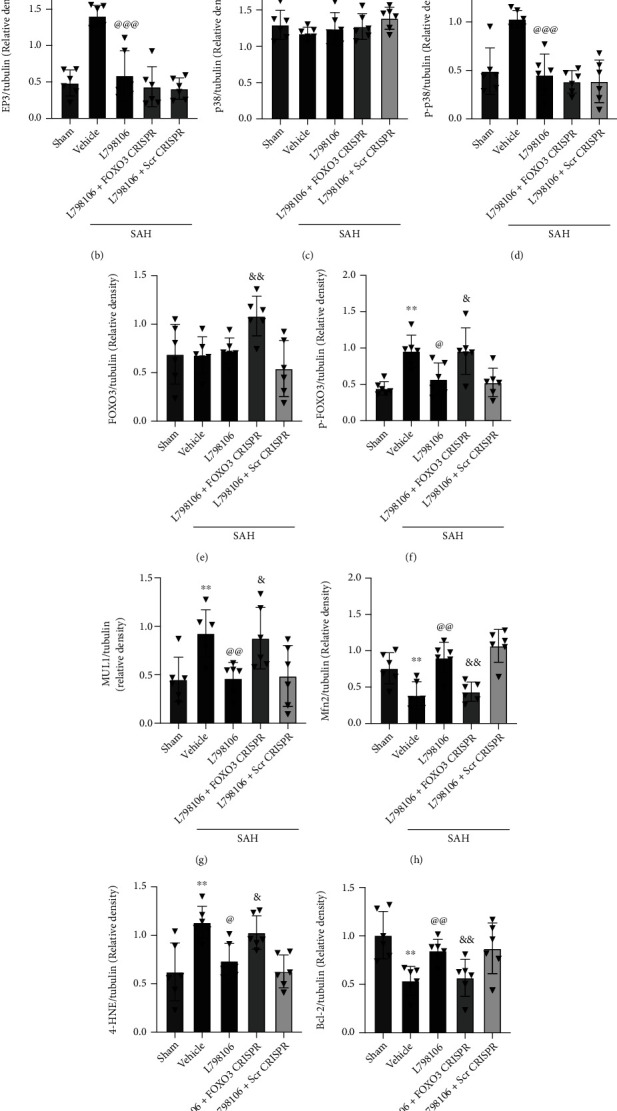
FOXO3 activation CRISPR abolished the beneficial effects of L798106 at 24 h after SAH. (a) Representative western blot bands and (b–l) quantification of EP3, p38, p-p38, FOXO3, p-FOXO3, Mul1, Mfn2, 4-HNE, Bcl-2, Bax, and CC3. Vehicle, 10% DMSO; Scr CRISPR, scramble CRISPR. ^∗∗^*p* < 0.01 and ^∗∗∗^*p* < 0.001 vs. sham group; ^@^*p* < 0.05, ^@@^*p* < 0.01, and ^@@@^*p* < 0.001 vs. SAH + vehicle group. ^&^*p* < 0.05, ^&&^*p* < 0.01, and ^&&&^*p* < 0.001 vs. SAH + L798106 + Scr CRISPR group. Data was expressed as mean ± SD, *n* = 6 per group. One-way ANOVA, Tukey's post hoc test.

**Figure 11:**
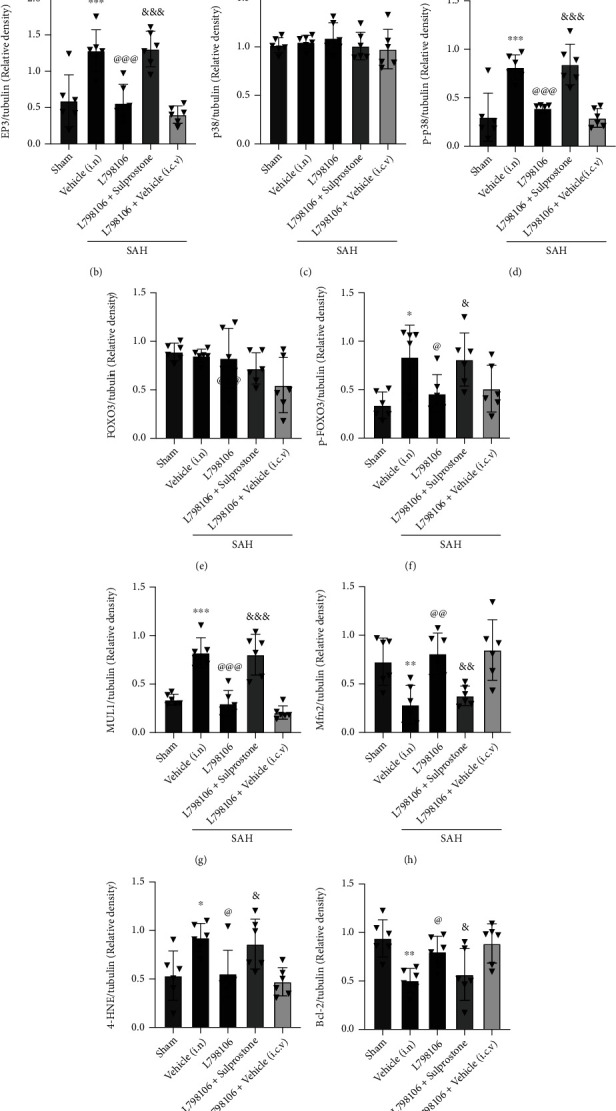
EP3 agonist sulprostone abolished the beneficial effects of L798106 at 24 h after SAH. Representative western blot bands (a) and quantification of EP3, p38, p-p38, FOXO3, p-FOXO3, Mul1, Mfn2, 4-HNE, Bcl-2, Bax, and CC3 (b–l). Vehicle, 10% DMSO; Scr CRISPR, scramble CRISPR. ^∗^*p* < 0.05, ^∗∗^*p* < 0.01, and ^∗∗∗^*p* < 0.001 vs. sham group; ^@^*p* < 0.05, ^@@^*p* < 0.01, and ^@@@^*p* < 0.001 vs. SAH + vehicle group. ^&^*p* < 0.05, ^&&^*p* < 0.01, and ^&&&^*p* < 0.001 vs. SAH + L798106 + Scr CRISPR group. Data was represented as mean ± SD, *n* = 6 per group. One-way ANOVA, Tukey's post hoc test.

Western blot results revealed SAH remarkably upregulated the protein level of EP3, p-p38, p-FOXO3, Mul1, 4-HNE, Bax, and cleaved caspase-3 but decreased the protein levels of Mfn2 and Bcl-2 compared to the sham group. The inhibition of EP3 by L798106 decreased the expression of EP3, p-p38, p-FOXO3, MUL1, 4-HNE, Bax, and cleaved caspase-3 but upregulated the expression of Mfn2 and Bcl-2 in SAH rats (Figures 10 and 11). Pretreatments of FOXO3 activation CRISPR (Figures 10(a)–10(l)) or EP3 agonist sulprostone (Figures 11(a)–11(l)) reversed the antiapoptotic and antioxidative stress effects of L798106 in SAH rats.

## 4. Discussion

In present study, we explored the effect of EP3 inhibition on oxidative stress, neuronal apoptosis, and related signaling pathway in a rat model of SAH. The novel findings are as follows: (1) the brain protein level of PGES2 (a proxy of PGE2) was increased at 3 h and peaked at 12 h post-SAH; the protein levels of EP1, EP2, EP3, EP4, and Mul1 were increased as early as 6 h and peaked at 24 h after SAH, in which the extent of EP3 increase was the most; (2) EP3 was mainly expressed in neurons, and a small amount was expressed in astrocytes and microglia at 24 h after SAH; (3) EP3 inhibition with L798106 at a dosage of 180 *μ*g/kg or EP3 knockout with EP3 KO CRISPR improved neurological outcome in SAH rats at 24 h after SAH. (4) L798106 or EP3 KO CRISPR attenuated neuronal apoptosis and oxidative stress at 24 h post-SAH. Such effects were persistent to 7 d after SAH. (5) EP3 inhibition with L798106 improved long-term neurological deficits and neuronal degeneration at 28 d after SAH. (6) EP3 inhibition with L798106 ameliorated oxidative stress injury and neuronal apoptosis through p38/FOXO3/Mul1/Mfn2 pathway.

Oxidative stress and neuronal apoptosis are the major pathological processes occurred in EBI after SAH [10]. Previous studies demonstrated that PGE2 exaggerated the brain injury and apoptotic neuronal death after ischemic stroke [52]. Among the four identified prostaglandin receptors (EP1-4) for prostaglandin E2 (PGE2), EP3 is reported to be the most abundantly expressed PGE2 receptor subtype in the brain [14]. A few studies suggested that EP3 was predominantly expressed in neurons under normal condition, and it was expressed in microglia and astrocytes pathologically [53]. The upregulation of the EP3 expression was reported in several types of brain injury models including ischemic stroke, intracerebral hemorrhage, and traumatic brain injury [16, 20, 52, 54–57]. PGE2-EP3 signaling axis plays an important role in modulating brain injury, inflammation, and neurologic functional recovery after ICH or ischemia stroke [16, 20, 21, 54, 56]. EP3 inhibition improved oxidative stress injury and apoptosis [21, 37, 58–61]. We consistently demonstrated that the endogenous expression of PGE2 and its receptors EP1-4 were increased in the ipsilateral brain hemisphere after SAH. The increase of EP3 was the most, and this receptor mainly localized on neurons. The result suggests that PGE2-EP3 signaling may contribute to the neuronal pathology after SAH. In addition, mitofusin2 (Mfn2) is a mitochondrial dynamin-related protein involved in the mitochondrial fusion reaction. The downregulation of Mfn2 by Mul1 exacerbated mitochondrial dysfunction and cell death after ischemic stroke [33]. Mfn2 could attenuate mitochondrial damage, cellular oxidative stress, and apoptosis in the animal models of cerebral ischemia-reperfusion (IR) injury and Alzheimer disease [62–64]. In our study, there was an increase in the endogenous Mfn2 along with EP3, suggesting the involvements of Mfn2 in PGE2-EP3 signaling after SAH.

L798106 is a potent and highly selective prostaglandin E2 Receptor EP3 antagonist that blocks the ligand binding to EP3 receptor, this inhibiting the activation [65]. The downregulatory effect of L798106 on EP3 protein levels was also found in hepatocellular carcinoma cells [66], tuberous sclerosis complex(TSC) cells [67], and breast cancer cells [68]. Although the specific protective mechanism of EP3 inhibition remains unclear, recent research has been focusing on the effects of antioxidative stress and antiapoptosis. EP3 activation exacerbated neuronal apoptosis after the experimental ICH [21]. EP3 antagonist protected against brain injury through the anti-inflammatory and antiapoptotic effects in the transient focal ischemia model [20]. The EP3 inhibition decreased NAPDH oxidase expression/activity and increased mitochondrial membrane potential in vascular smooth muscle cells [69]. The EP3 deletion reduced proinflammatory gene expression, cytokine production, and oxidative stress in an Alzheimer disease model [60]. Taking together, these studies suggested that L798106 treatment provided neuroprotection by reducing oxidative stress and neuronal apoptosis.

In our present study, intranasal treatment with L798106 1 h post-SAH downregulated the EP3 expression and ameliorated short-term and long-term neurological deficits. The assessments of 8-OHdG, TUNEL, and FJC revealed the reduction of oxidative stress levels and neuronal apoptosis in SAH rats treated by either L798106 or EP3-knockout CRISPR at 24 h or 7 d after SAH induction. Consistently, L798106 treatment significantly increased the brain expression of Bcl-2 but decreased the brain expression of 4-HNE, Bax, and cleaved caspase-3 at 24 h post-SAH. Thus, the results suggested that the inhibition of EP3 with L798106 improved neurological deficits post-SAH likely by attenuating oxidative stress injury and neuronal apoptosis.

We further investigated the molecular mechanisms underlying EP3 inhibition-mediated antioxidative stress and antineuronal apoptosis after of SAH. Previous study showed that the EP3 activation promoted endothelial cell apoptosis by enhancing p38 MAPK phosphorylation and decreasing Bcl-2 expression in cultured human umbilical vein endothelial cells [37]. Phosphorylation of FOXO3 on Ser-7 by p38 is essential for its nuclear relocalization in response to treatment in breast carcinoma cells [70]. FOXO3 activation can increase the transcription of Mul1, which ubiquitinates and degrades Mfn2 and leads to mitochondrial fragmentation [71]. The downregulation of Mfn2 by Mul1 increased the fragmented mitochondria concomitant with mitochondrial dysfunction and cell death in ischemic stroke [33]. We found that the EP3 inhibition with L798106 remarkably decreased the expression of p-p38, p-FOXO3, Mul1, 4-HNE, Bax, and cleaved caspase-3 in the brain tissue at 24 h post-SAH. The activation of EP3 upregulated p-p38 expression and its downstream signaling molecules of p-FOXO3, Mul1, 4HNE, Bax, and cleaved caspase-3 but downregulated the expression of Mfn2 and Bcl-2. Furthermore, to examine potential downstream mediators of EP3, we examined the effect of increased expression of activated FOXO3 following the administration of FOXO3 activation CRISPR. The activation of FOXO3 had no effects on p38 phosphorylation, but it remarkably downregulated the protein levels of Mfn2 and Bcl-2, and upregulated the protein level of Mul1, 4-HNE, Bax, and cleaved caspase-3. The results demonstrated that EP3 inhibition attenuated the oxidative stress and neuronal apoptosis post-SAH partly by u-regulating the Mfn2 expression via suppressing p-p38/p-FOXO3/Mul1 signaling pathway.

This study has several limitations. Firstly, although we focused on neurons in the current study, the anti-inflammation and blood-brain barrier (BBB) preservation may also participate in the overall neurological benefits of EP3 inhibition. Secondly, EP3 activation promotes apoptosis through a variety of signaling pathways such as calcium/CamKII/Erk, TGF-*β*/Smad, and cAMP [72–74]. Future studies are needed to investigate the underlying molecular mechanisms other than p-p38/p-FOXO3/Mul1/Mfn2 signaling. Thirdly, the gender difference was not addressed, in which only male rats were used. Fourthly, PGE2 polyclonal Ab from Bioss Inc. was used to measure PGE2 level indirectly. It reacts with isomerase that catalyze the conversion of PGH2 into more stable PGE2. The level of isomerase is an indirect indicator of PGE2 level in brain tissue. ELISA validation is necessary in future experiments. Lastly, while L798106 is a highly specific EP3 antagonist, it is important to note that sulprostone is not as specific as an agonist for EP3. Since sulprostone is only about 100-fold more specific for EP3 than EP1, the effect in this experiment does not rule out targeting EP1, although the most dramatic changes in EP3 are shown in our data.

## 5. Conclusions

In conclusion, we demonstrated the inhibition of EP3 with L798106 ameliorated neurological impairment by reducing oxidative stress and neuronal apoptosis post-SAH in rats. These protective effects were, at least in part, via the p-p38/p-FOXO3/Mul1/Mfn2 signaling pathway. EP3 may serve as a potential therapeutic target for SAH patients.

## Data Availability

The data used to support the findings of this study are available from the corresponding authors upon request.

## Supplementary Materials

Supplementary Materials Additional file 1. Figure s1: schematic illustration of the experimental design. Additional file 2. Figure s2: Efficacy of EP3 knock out CRISPR and FOXO3 activation CRISPR on EP3 and p-FOXO3 expression. Representative western blot bands (A, B) and densitometric quantification of EP3 (C) and pFOXO3 (D). scr CRISPR, scramble CRISPR; KO CRISPR, knockout CRISPR; ACT CRISPR, activation CRISPR. ^∗∗^*p* < 0.01 vs. naive group + scr CRISPR group; ^@@^*p* < 0.01 vs. SAH + scr CRISPR group. Data was expressed as mean ± SD, *n* = 6 per group. One-way ANOVA, Tukey's post hoc test.
