# Supplementary material for: Inhibition of Prostaglandin E2 Receptor EP3 Attenuates Oxidative Stress and Neuronal Apoptosis Partially by Modulating p38MAPK/FOXO3/Mul1/Mfn2 Pathway after Subarachnoid Hemorrhage in Rats
Source: Oxid Med Cell Longev. 2022 Dec 9;2022:7727616. doi: 10.1155/2022/7727616 (PMC9757947; doi:10.1155/2022/7727616)
Supplement: Supplementary Materials — Additional file 1. Figure s1: schematic illustration of the experimental design. Additional file 2. Figure s2: Efficacy of EP3 knock out CRISPR and FOXO3 activation CRISPR on EP3 and p-FOXO3 expression. Representative western blot bands (A, B) and densitometric quantification of EP3 (C) and pFOXO3 (D). scr CRISPR, scramble CRISPR; KO CRISPR, knockout CRISPR; ACT CRISPR, activation CRISPR. ∗∗p < 0.01 vs. naive group + scr CRISPR group; @@p < 0.01 vs. SAH + scr CRISPR group. Data was expressed as mean ± SD, n = 6 per group. One-way ANOVA, Tukey's post hoc test. [file 7727616.f1.docx]

**Figure. s1 Experimental design.**

**Figure. s2 Efficacy of EP3 knock out CRISPR and FOXO3 activation CRISPR on EP3 and p-FOXO3 expressions.** Representative western blot bands (A), (B) and densitometric quantification of EP3 (C) and pFOXO3 (D). scr CRISPR, scrambled CRISPR; KO CRISPR, Knockout CRISPR; ACT CRISPR, activation CRISPR. ***p* < 0.01 vs. Naïve group + scr CRISPR group; ^@@^*p* < 0.01 vs. SAH + scr CRISPR group. Data was expressed as mean ± SD, n = 6 per group. One-way ANOVA, Tukey's post hoc test.
